# An Object Detection Method Based on Frequency-Band Enhancement and Multi-Scale Fusion

**DOI:** 10.3390/jimaging12090447

**Published:** 2026-09-16

**Authors:** Zhenzhao Dai, Yongsheng Qiu, Yuanyao Lu

**Affiliations:** 1 School of Electrical and Control Engineering, North China University of Technology, Beijing 100144, China; dzz970703@163.com; 2 School of Computer Science and Technology, Kashi University, Kashi 844000, China; qys15840212165@163.com; 3 School of Artificial Intelligence and Computer Science, North China University of Technology, Beijing 100144, China

**Keywords:** object detection, RT-DETR, wavelet transform, feature fusion, autonomous driving

## Abstract

Although Transformer-based real-time object detectors have achieved promising performance in autonomous driving scenarios, their ability to detect small objects remains limited. This limitation primarily arises because small objects occupy only a few pixels in an image and contain weak edge and texture information, which can be further degraded during feature extraction and multiscale feature propagation. To address these issues, this study proposes a wavelet-based frequency-aware feature enhancement method using RT-DETR as the baseline network. First, a Wavelet Frequency Unit is introduced into the feature fusion stage of the RT-DETR neck. The unit employs the Haar wavelet transform to decompose the input features into low- and high-frequency subbands, thereby decoupling information across different frequency components. Second, residual enhancement and a frequency attention mechanism are applied to strengthen edge and texture details in the high-frequency branch. Finally, the low-frequency subband is fused with low-resolution features across scales, followed by feature reconstruction using the inverse wavelet transform. This design improves the representation of small objects in the feature space. Tests on KITTI and BDD100K verify the method. On KITTI, it obtains 95.5% mAP@0.5 and 69.7% mAP@0.5:0.95, exceeding the RT-DETR baseline by 1.8 and 1.1 percentage points. APs and ARs rise by 2.7 and 2.3 percentage points. On the selected BDD100K subset, the corresponding mAP@0.5 and mAP@0.5:0.95 values are 51.7% and 29.7%.

## 1. Introduction

With the continuous advancement of intelligent transportation systems and autonomous driving technologies, object detection has become a fundamental task in autonomous perception systems [1,2,3]. These systems must accurately and efficiently identify various road elements, including vehicles, pedestrians, cyclists, traffic signs, and other objects, to support environmental understanding, motion planning, and safety-related decision-making. In recent years, Transformer-based object detection approaches have attracted considerable attention in computer vision due to their strong feature representation capability [4,5,6,7]. By exploiting self-attention mechanisms, Transformer detectors can capture long-range dependencies among image features and achieve end-to-end object classification and bounding-box regression. Among these approaches, DETR [8] introduced a pioneering Transformer-based framework for object detection. By modeling global interactions between different image regions, DETR removes the dependence on manually designed components and reduces the complex post-processing procedures required by traditional detectors. To improve detection efficiency for real-time applications, RT-DETR [9] further enhances the DETR architecture by integrating an efficient hybrid encoder. This improvement accelerates inference while maintaining competitive detection accuracy, making RT-DETR more suitable for practical autonomous driving scenarios.

**Figure 1:**
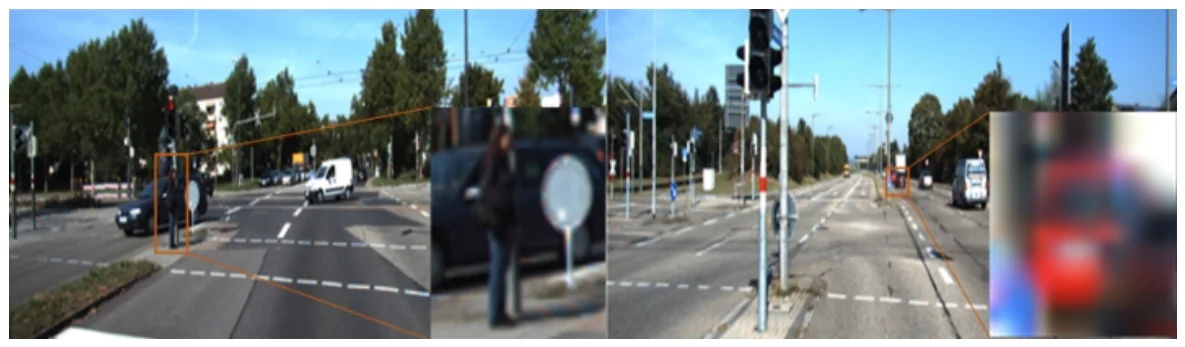
Small-scale objects in autonomous driving scenarios and their corresponding enlarged views.

Although RT-DETR achieves competitive performance in general object detection tasks, its capability for identifying small objects in autonomous driving scenarios remains insufficient [10,11]. As shown in Figure 1, small targets usually occupy only a limited number of pixels and contain weak edge, texture, and local structural information. These fine-grained features are highly vulnerable to information loss during feature extraction, downsampling, and cross-scale feature propagation. Moreover, object occlusion, illumination changes, and complex background interference can further reduce the discriminability between target features and surrounding environmental information, thereby increasing the probability of missed detections and false positives.

Existing multi-scale feature fusion strategies mainly focus on directly combining or aggregating spatial-domain features without considering the intrinsic characteristics of different frequency components [12]. Consequently, these approaches may struggle to effectively preserve detailed structural information of small objects. Unlike conventional spatial-domain fusion methods, wavelet transformation decomposes input features into low-frequency structural components and high-frequency detail components [13]. The low-frequency branch mainly preserves global semantic and structural information, while the high-frequency branch captures fine-grained details, such as object boundaries, textures, and local contours. Therefore, introducing frequency-domain feature modeling into the fusion process provides a potential solution for alleviating the degradation of small-object representations during feature transmission.

Motivated by the above observations, this study develops a frequency-aware feature enhancement framework based on RT-DETR. Specifically, a Wavelet Frequency Unit (WFU) is incorporated into the multi-scale feature fusion stage of the RT-DETR neck. Through wavelet decomposition, the WFU separates structural information from high-frequency details and performs targeted enhancement on high-frequency components and low-frequency cross-scale features. This strategy improves the representation capability of object boundaries, textures, local contours, global structures, and contextual semantics, thereby enhancing the detection performance of small-scale objects.

The main contributions of this study are summarized as follows:

1. A frequency-band enhancement and multi-scale fusion framework based on RT-DETR is introduced for object detection. To overcome the insufficient representation capability of small objects caused by limited pixel information, weak edges, and texture loss in autonomous driving scenarios, a frequency-aware feature enhancement strategy is integrated into the RT-DETR neck to improve small-object feature representation.
2. A Wavelet Frequency Unit (WFU) is developed to decompose high-resolution feature maps into low-frequency structural components and high-frequency detail components through Haar wavelet transformation. This decomposition enables the separation of semantic structures and fine-grained details, reducing the loss of small-object information during conventional spatial-domain feature fusion.
3. A dual-path enhancement strategy combining high-frequency detail refinement and low-frequency cross-scale fusion is proposed. In the high-frequency branch, residual enhancement and frequency attention mechanisms are employed to strengthen edge and texture representations of small objects. In the low-frequency branch, deeper semantic features are fused to improve the joint representation of object structures and contextual information.
4. Extensive experiments are conducted on the KITTI and BDD100K datasets. Comparative experiments, ablation studies, and repeated evaluations are performed on the KITTI dataset, while small-object detection assessments and visualization analyses are conducted on both datasets to comprehensively validate the effectiveness and robustness of the proposed approach.

## 2. Related Work

This study adopts RT-DETR as the baseline detector and enhances its feature fusion capability by introducing wavelet-based feature decomposition. This section provides an overview of Transformer-based object detection approaches and discusses the application of wavelet transforms in visual recognition, establishing the theoretical motivation for the proposed framework.

### 2.1. Transformer-Based Object Detection Methods

Transformer-based object detection methods have emerged as an important research direction in the field of object detection. DETR formulates object detection as a set prediction problem and directly predicts object categories and bounding boxes through learnable object queries. This end-to-end paradigm eliminates several manually designed components used in traditional detectors, such as anchor generation, proposal extraction, and non-maximum suppression, thereby simplifying the overall detection pipeline. However, the original DETR relies heavily on global attention for feature interaction, resulting in slow convergence during training. Moreover, the extracted high-level semantic features often lack sufficient fine-grained details, which limits its performance in detecting small and multi-scale objects.

To improve the training efficiency and scale adaptability of DETR, Deformable DETR [14] introduces deformable attention, which performs feature interaction at a small set of key sampling locations on multiscale feature maps, thereby reducing the computational cost associated with global attention. DAB-DETR [15] represents object queries as dynamic anchor boxes, providing explicit positional priors for bounding-box prediction. DINO [16] improves the matching process through denoising training and query initialization, thereby alleviating training instability. Co-DETR [17] introduces a collaborative supervision mechanism to strengthen encoder feature learning and further improve detection performance.

In practical deployment scenarios, Transformer-based detectors often incur considerable computational overhead. RT-DETR simplifies the encoder and multiscale feature interaction process to reduce redundant computation, enabling end-to-end detection to meet real-time requirements. RT-DETRv2 [18] and LW-DETR [19] further reduce model complexity through improved training strategies and lightweight architectural designs. Although these methods enhance detection efficiency, their ability to exploit fine-grained information, such as edges and textures, remains limited.

The detection of small objects and objects embedded in complex backgrounds often depends heavily on local cues, including edges and textures. Although multiscale feature fusion enhances the complementary representation of features at different levels, fine-grained information is still susceptible to degradation during feature downsampling and cross-layer propagation. To address this limitation, the present study introduces a frequency-band enhancement mechanism into a Transformer-based detection framework and combines it with multiscale feature fusion to improve the representation of object details and scale variations.

### 2.2. Applications of Wavelet Transforms in Object Detection

Frequency-domain information can complement spatial-domain feature representations and enhance edges, textures, and weak-response regions. Consequently, frequency-domain modeling has received increasing attention in small-object detection. To address the challenges posed by densely distributed small objects and complex backgrounds, Zhang et al. proposed FFC-YOLO [20], which incorporates a frequency-aware module into the feature fusion process to strengthen small-object representations and reduce missed detections and false positives. Subsequent studies have also integrated frequency-domain enhancement into existing YOLO-series detectors. For example, Zhao [21] proposed an adaptive weighting and frequency-domain enhancement fusion method to improve the discriminability of small-object regions in complex backgrounds.

In addition to YOLO-based detectors, frequency-domain information has also been incorporated into Transformer-based detection frameworks. Freq-DETR [22] employs dual spatial- and frequency-domain branches to extract local spatial features and global frequency information. It further performs intra-scale interactions between high- and low-frequency components and uses an attention-guided feature pyramid for multiscale feature fusion. Its primary emphasis is on spatial–frequency collaboration and intra-scale frequency interaction. Ge et al. proposed VMC-DETR [23], which employs a frequency-domain heat conduction mechanism in the backbone to enhance local high-frequency textures. Multiscale feature aggregation and contextual attention are further used to improve information exchange among features at different scales. To address noise interference and rotated-object detection in remote sensing imagery, Yang et al. proposed WDARFNet [24]. This method uses the discrete wavelet transform to decompose features into different frequency components and combines channel selection, spatial selection, and multi-branch convolution to dynamically adjust the receptive field while selectively suppressing noisy high-frequency components.

Overall, existing frequency-domain detection methods improve small-object feature representations through spatial–frequency collaboration, high-frequency texture enhancement, frequency selection, and multiscale feature aggregation. However, most of these methods primarily emphasize the enhancement of local high-frequency information and pay insufficient attention to the complementary relationship between high-frequency details and low-frequency structural information. To address this issue, the proposed method introduces a discrete-wavelet-based frequency-band decoupling mechanism into the multiscale feature fusion stage of RT-DETR. The input features are decomposed into low-frequency structural components and directional high-frequency detail components. High-frequency detail enhancement and low-frequency cross-scale fusion are then performed separately, followed by feature reconstruction using the inverse wavelet transform. This design strengthens the joint representation of local details and global structures.

## 3. Method

This section presents the proposed detection network and its implementation, with particular emphasis on the overall network architecture and the design of the Wavelet Frequency Unit. To address the degradation of local structural and texture information associated with small objects during feature propagation, RT-DETR is adopted as the baseline network, and a frequency-aware feature enhancement module is introduced into its neck. By integrating high- and low-frequency feature decomposition, high-frequency detail enhancement, and low-frequency cross-scale fusion, the proposed method strengthens the joint representation of edge and texture details and global structural information.

### 3.1. Overall Network Architecture

**Figure 2:**
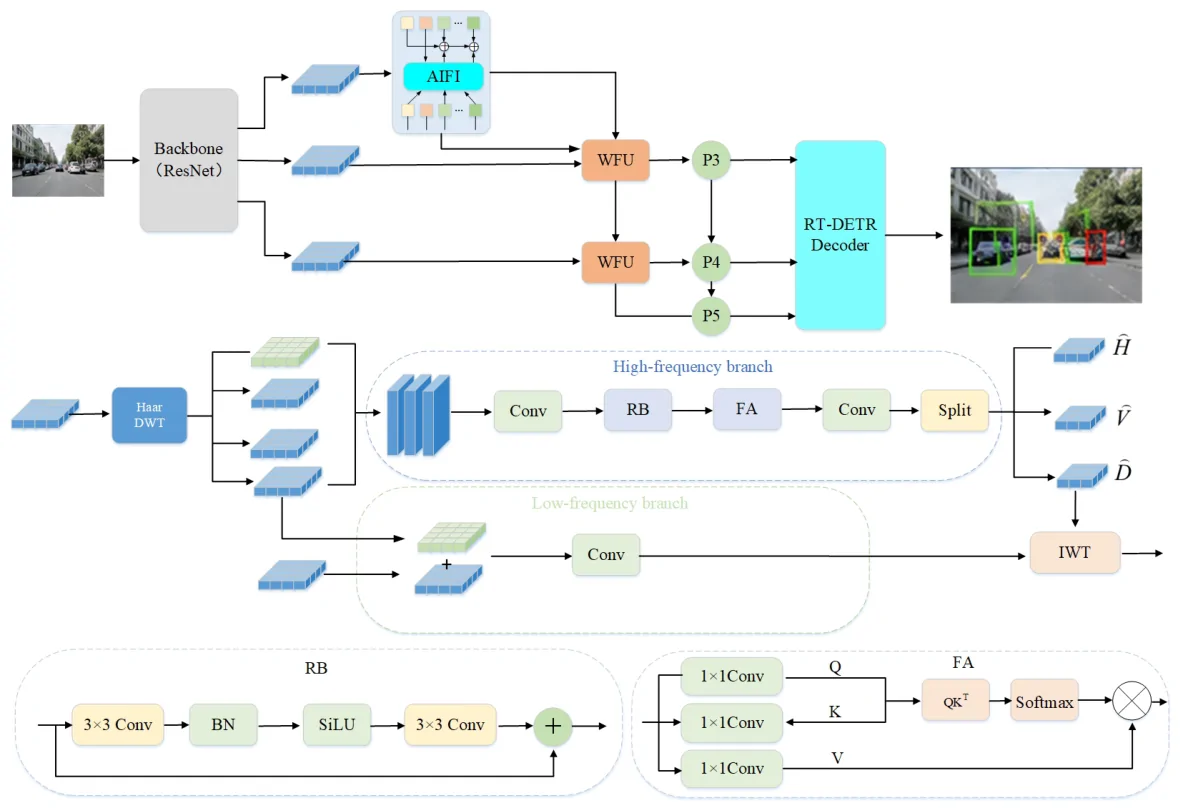
Architecture of the proposed improved model.

To address the degradation of detailed information during cross-scale feature propagation in RT-DETR, this study develops an object detection network based on frequency-band enhancement and multiscale fusion, as illustrated in Figure 2. The proposed network is built upon RT-DETR and consists of a ResNet backbone [25], an improved neck, and the RT-DETR decoder. The backbone extracts hierarchical features at different spatial resolutions, whereas the decoder retains the original query decoding and prediction mechanisms to perform object classification and bounding-box regression. The primary modifications are introduced into the multiscale feature fusion stage of the neck.

Within the neck, the highest-level feature is first processed by the Attention-based Intra-scale Feature Interaction (AIFI) module for global semantic modeling and is subsequently propagated to the intermediate- and low-level features through a top-down pathway. To mitigate the loss of edge and texture information during this semantic propagation process, two Wavelet Frequency Units (WFUs) are introduced at the cross-scale fusion stages. These units are used to fuse the high-level and intermediate-level features and the intermediate-level and low-level features, respectively. At each fusion stage, the lateral feature with a relatively high spatial resolution at the current scale is denoted by $X_{B}$, whereas the low-resolution semantic feature propagated from the deeper network layers is denoted by $Xs\in R^{B\times C_{S}\times H_{S}\times W_{S}}$.

Unlike conventional fusion strategies that directly align feature resolutions and concatenate feature channels, the WFU first applies Haar wavelet decomposition to $X_{B}$, producing one low-frequency subband and three directional high-frequency subbands. The high-frequency branch enhances detailed information such as object edges and textures, whereas the low-frequency branch incorporates high-level semantic information from $X_{S}$ The processed frequency components are then reconstructed into a spatial-domain feature through the inverse wavelet transform. In this manner, the WFU preserves local structural information at the current scale while integrating high-level semantic information from deeper layers.

The reconstructed features generated by the two WFUs are subsequently fed into the bottom-up path aggregation network (PAN) to strengthen information exchange among features at different scales. Finally, the resulting multiscale features are passed to the RT-DETR decoder for object classification and localization.

### 3.2. High-Frequency Detail Enhancement

The edges, textures, and local contours of small objects are susceptible to degradation during feature downsampling and cross-scale propagation. To preserve such fine-grained information, a high-frequency enhancement branch is incorporated into the WFU to jointly model directional high-frequency wavelet responses.

Given a high-resolution input feature $X_{b}\in R^{B\times C\times H\times W}$, a two-dimensional Haar wavelet transform [26] is applied to decompose it into a low-frequency subband *a* and three high-frequency subbands, *h*, *v*, and *d*, corresponding to the horizontal, vertical, and diagonal directions, respectively:

(1) $$\left(a,h,v,d\right)=\mathrm{DWT}\left(X_{b}\right),$$

The discrete wavelet transform is implemented using four fixed 2×2 Haar filters through channel-wise grouped convolution. The convolution stride is set to 2, no padding is applied, and the filter coefficients remain fixed throughout training. Because the spatial dimensions of the input features are even, the dimensions of the decomposed subbands are

(2) $$a,h,v,d\in R^{B\times C\times H^{\prime }\times W^{\prime }},\;H^{\prime }=\frac{H}{2},\;W^{\prime }=\frac{W}{2}$$

The low-frequency subband *a* primarily preserves global structural information, whereas the three high-frequency subbands encode directional edge and texture responses.

To prevent premature mixing of directional information caused by direct summation, the three high-frequency subbands are concatenated along the channel dimension:

(3) $$F_{H}=\mathrm{Cat}\left(h,v,d\right)$$

Subsequently, a 1×1 convolution is used to compress the number of channels from 3C to *C*, after which the feature is processed sequentially by the residual enhancement block and the high-frequency subband spatial attention module. The enhanced feature is then projected back to 3C channels and fused with the original joint high-frequency feature through a residual connection:

(4) $${\tilde{F}}_{H}=F_{H}+\phi_{e}\left(FA\left[RB\left(\phi_{r}\left(F_{H}\right)\right)\right]\right)$$

where $\phi_{r}:3C\to C$ and $\phi_{e}:C\to 3C$ denote the channel-compression and channel-expansion operations, respectively. Both operations are implemented using 1×1 convolutions with a stride of 1 and no padding. Channel compression controls the computational cost of high-frequency feature modeling, whereas the outer residual connection preserves the original directional responses. Finally, the enhanced joint high-frequency feature is evenly split into three subbands along the channel dimension:

(5) $$\left(\hat{h},\hat{v},\hat{d}\right)=\mathrm{Split}\left({\hat{F}}_{H}\right)$$

where $\hat{h}$, $\hat{v}$, and $\hat{d}$ have the same dimensions, $B\times C\times H^{\prime }\times W^{\prime }$. The three enhanced high-frequency subbands are subsequently used for feature reconstruction through the inverse Haar wavelet transform.

#### 3.2.1. Residual Enhancement Block

Let the channel-compressed high-frequency feature be defined as:

(6) $$Z=\phi_{r}\left(F_{H}\right)\in R^{B\times C\times H^{\prime }\times W^{\prime }}$$

The residual enhancement block consists of two 3×3 convolutional layers. Both layers have *C* input and output channels, a stride of 1, and a padding size of 1; therefore, the spatial dimensions of the feature remain unchanged. The first convolutional layer is followed by batch normalization and a SiLU activation function, whereas the second convolutional layer does not use normalization or an activation function. The computation is expressed as follows:

(7) $$F_{\mathrm{RB}}=Z+{\mathrm{Conv}}_{3\times 3}^{\left(2\right)}\left[\delta \left(\mathrm{BN}\left({\mathrm{Conv}}_{3\times 3}^{\left(1\right)}\left(Z\right)\right)\right)\right]$$

where δ(⋅) denotes the SiLU activation function. The convolutional operations capture local variations in edges and textures, whereas the residual connection preserves the original high-frequency information and stabilizes feature optimization. The module output satisfies:

(8) $$F_{\mathrm{RB}}\in R^{B\times C\times H^{\prime }\times W^{\prime }}$$

#### 3.2.2. High-Frequency Subband Spatial Attention

Local convolutional operations may be insufficient to capture spatially distant high-frequency responses. Therefore, a high-frequency subband spatial attention module is introduced after the residual enhancement block to model spatial correlations within the wavelet high-frequency features. This module operates on the spatial responses of the high-frequency subbands rather than directly assigning weights to Fourier-frequency components.

Given the input feature $F_{\mathrm{RB}}\in R^{B\times C\times H^{\prime }\times W^{\prime }}$, three 1×1 convolutions are used to generate the query, key, and value features:

(9) $$Q=\varphi_{q}\left(F_{\mathrm{RB}}\right),\;K=\varphi_{k}\left(F_{\mathrm{RB}}\right),\;V=\varphi_{v}\left(F_{\mathrm{RB}}\right)$$

where all three convolutions have *C* input and output channels, a stride of 1, and no padding. Let $N=H^{\prime }W^{\prime }$ The three features are then reshaped as follows:

(10) $$Q,V\in R^{B\times N\times C},\;K\in R^{B\times C\times N}$$

The attention matrix and the output feature are computed as follows:

(11) $$A=\mathrm{Softmax}\left(\frac{QK}{\sqrt{C}}\right),\;F_{\mathrm{FA}}=\mathrm{Reshape}\left(AV\right)$$

where, $A\in R^{B\times N\times N}$, The Softmax operation is applied along the spatial-position dimension, and the scaling factor $\frac{1}{\sqrt{C}}$ controls the numerical range of the dot-product values. The attention output is subsequently reshaped as: $F_{FA}\in R^{B\times 3C\times H^{\prime }\times W^{\prime }}$, After channel expansion and residual fusion, ${\overset{⌢}{F}}_{H}$ has $B\times 3C\times H^{\prime }\times W^{\prime }$ channels. Following the original ordering of the high-frequency subbands, ${\overset{⌢}{F}}_{H}$ is split along the channel dimension as follows:

(12) $$\left(\hat{h},\hat{v},\hat{d}\right)=\mathrm{Split}\left({\hat{F}}_{H}\right)$$

where $\overset{⌢}{h}$, $\overset{⌢}{v}$, and $\overset{⌢}{d}$ all have dimensions $B\times C\times H^{\prime }\times W^{\prime }$. The three components serve as the horizontal, vertical, and diagonal high-frequency inputs, respectively, for the subsequent inverse Haar wavelet transform.

### 3.3. Multiscale Fusion and Feature Reconstruction

The high-frequency subbands primarily characterize object edges and textures, whereas the low-frequency subband preserves global contours and structural information. To supplement the low-frequency branch with deep semantic information, the low-frequency subband is further fused with the low-resolution semantic feature. Let the low-resolution input feature be defined as:

(13) $$X_{s}\in R^{B\times C_{s}\times H_{s}\times W_{s}}$$

First, nearest-neighbor interpolation is applied to resize its spatial dimensions to $H^{\prime }\times W^{\prime }$, matching those of the low-frequency subband *a*. The resized feature is then concatenated with *a* along the channel dimension. The concatenated feature is processed by two 1×1 convolutions for channel fusion. The first convolution is followed by batch normalization and a SiLU activation function, whereas the second convolution maps the number of output channels to *C*. The fused low-frequency subband is expressed as follows:

(14) $$\hat{a}=a+\phi_{l}\left[\mathrm{Cat}\left(a,\mathrm{Resize}(X_{s})\right)\right]$$

where $\phi_{l}\left(\cdot \right)$ denotes the low-frequency channel transformation operation, and $\overset{⌢}{a}$ has dimensions $B\times C\times H^{\prime }\times W^{\prime }$. The residual connection introduces contextual semantic information from the low-resolution feature while preserving the original low-frequency structure.

After high-frequency enhancement and low-frequency fusion, the fused low-frequency subband and the three enhanced high-frequency components are concatenated along the channel dimension in the order of low frequency, horizontal high frequency, vertical high frequency, and diagonal high frequency.

(15) $$F_{\mathrm{coef}}=\mathrm{Cat}\left(\hat{a},\hat{h},\hat{v},\hat{d}\right)$$

where $F_{coef}\in R^{B\times 4C\times H^{\prime }\times W^{\prime }}$. This channel ordering is consistent with the subband arrangement used in the forward Haar wavelet decomposition. Finally, spatial feature reconstruction is performed using the inverse Haar wavelet transform:

(16) $$F_{\mathrm{out}}=\mathrm{IWT}\left(F_{\mathrm{coef}}\right)$$

The inverse transform employs fixed 2×2 Haar synthesis filters matched to those used in the forward decomposition, with a stride of 2 and zero padding, thereby restoring the feature to its original spatial resolution, $F_{\mathrm{out}}\in R^{B\times C\times H\times W}$. Consequently, the directional details enhanced by the high-frequency branch and the structural semantics fused by the low-frequency branch are jointly reconstructed in the spatial domain and used as the output of the current cross-scale fusion stage.

## 4. Experiments

### 4.1. Datasets and Data Splits

The KITTI dataset [27] is a widely used public benchmark for visual perception research in autonomous driving. It was collected using an instrumented vehicle in real-world road environments and covers a variety of traffic scenarios, including urban streets, residential areas, rural roads, and highways. The dataset contains traffic participants at different scales, such as vehicles, pedestrians, and cyclists. The KITTI 2D object detection benchmark consists of 7481 training images and 7518 test images. The original annotations include nine categories: Car, Van, Truck, Pedestrian, Person_sitting, Cyclist, Tram, Misc, and DontCare. In this study, the Misc and DontCare categories are excluded, and Person_sitting is merged into the Pedestrian category. Consequently, six detection categories are retained: Car, Van, Truck, Pedestrian, Cyclist, and Tram.

The experimental dataset is constructed from the 7481 annotated training images, which are divided into training, validation, and test sets at a ratio of 8:1:1. Because data partitioning and random initialization may affect detection performance [28,29], all comparison models use the same data split to ensure a fair evaluation.

To evaluate the applicability of the proposed method across different data distributions and complex road environments, additional experiments are conducted on the BDD100K dataset [30]. Owing to limited computational resources, an experimental subset containing 20,955 annotated images is selected from BDD100K. These images are also divided into training, validation, and test sets at a ratio of 8:1:1.

Because this study primarily focuses on traffic objects that occupy relatively small image regions, the commonly used object-scale definition is adopted. Specifically, an object whose annotated bounding-box area is smaller than $32^{2}$ pixels is regarded as a small object [31].

### 4.2. Evaluation Metrics

Model performance is evaluated in terms of detection accuracy, model complexity, and inference efficiency. Detection accuracy is measured using precision (*P*), recall (*R*), ${\mathrm{AP}}_{50}$, and ${\mathrm{AP}}_{50:95}$. Small-object detection performance is evaluated using ${\mathrm{AP}}_{s}$ and ${\mathrm{AR}}_{s}$ Model complexity is quantified by the number of parameters, whereas inference efficiency is evaluated in frames per second (FPS). The average precision for small objects, ${\mathrm{AP}}_{s}$, and the average recall for small objects, ${\mathrm{AR}}_{s}$, are adopted as scale-specific evaluation metrics. ${\mathrm{AP}}_{s}$ is calculated for small objects over IoU thresholds ranging from 0.50 to 0.95 in increments of 0.05 [31]. It provides a comprehensive evaluation of the model’s classification and localization performance for small objects. Similarly, ${\mathrm{AR}}_{s}$ is calculated using the same object-scale definition and IoU threshold range and measures the model’s overall ability to detect small objects. Based on the matching results between model predictions and ground-truth annotations, precision and recall are defined as follows:

(17) $$\begin{array}{cc} P & =\frac{TP}{TP+FP},\\ R & =\frac{TP}{TP+FN}. \end{array}$$

where TP, FP, and FN denote the numbers of true positives, false positives, and false negatives, respectively. Precision reflects the reliability of the predicted detections, whereas recall measures the proportion of ground-truth objects successfully detected by the model.

(18) $${\mathrm{AP}}_{i}=\int_{0}^{1}P_{i}\left(R\right)\;dR$$

where $AP_{i}$ represents the average precision of the i-th category. Assuming that the dataset contains C detection categories, the mean average precision (mAP) is calculated as follows:

(19) $$\mathrm{mAP}=\frac{1}{C}\sum_{i=1}^{C}{\mathrm{AP}}_{i}$$

Specifically, mAP0.5 denotes the mean average precision across all categories under an IoU threshold of 0.5, which mainly evaluates the model’s object detection and classification performance. mAP0.5:0.95 computes the average mAP over IoU thresholds from 0.50 to 0.95 with a step size of 0.05, providing a more comprehensive assessment with stricter requirements on bounding box localization accuracy.

The number of parameters represents the total number of learnable parameters in the model and is used to characterize the model complexity. FPS indicates the number of images processed by the model per second and is adopted to evaluate the inference speed. All models are evaluated under the same input resolution, data split, and testing environment to ensure the comparability of experimental results.

To evaluate the stability of multiple training runs, this study employs the mean, sample standard deviation, and 95% confidence interval for statistical analysis. Assuming that the model is trained independently *n* times and the result of the *i*-th experiment is $x_{i}$, the mean and sample standard deviation are calculated as follows:

(20) $$\begin{array}{cc} \bar{x} & =\frac{1}{n}\sum_{i=1}^{n}x_{i},\\ s & =\sqrt{\frac{1}{n-1}\sum_{i=1}^{n}{\left(x_{i}-\bar{x}\right)}^{2}}. \end{array}$$

Considering the limited number of repeated experiments, the 95% confidence interval of the mean is calculated based on the Student’s t-distribution:

(21) $${\mathrm{CI}}_{95\%}=\bar{x}\pm t_{0.975,n-1}\frac{s}{\sqrt{n}}$$

where $t_{0.975,n-1}$ represents the two-tailed critical value of the Student’s t-distribution with n−1 degrees of freedom. The standard deviation is used to reflect the dispersion of results across different training runs, while the confidence interval characterizes the uncertainty of the estimated average performance.

### 4.3. Experimental Settings

The experiments were conducted on the Ubuntu 18.04 operating system. Model training and testing were implemented based on the PyTorch 1.12.1 deep learning framework. To reduce the impact of environmental variations on the experimental results, all models were trained and evaluated under the same hardware and software configurations.

During the training and validation stages, the input image size was uniformly set to 640 × 640, and the batch size was set to 16. All models were trained for 300 epochs, with an initial learning rate of 0.005 and a final learning rate set to 0.01 times the initial learning rate. The AdamW optimizer [32] was adopted, with the weight decay coefficient set to 0.0005. A warmup strategy was employed during training, with the warmup epoch set to 3, the initial warmup momentum set to 0.8, and the warmup bias learning rate set to 0.1. The bounding box regression loss weight was set to 7.5, while the remaining training parameters were kept consistent with those of the baseline model.

### 4.4. Comparative Experiments

To verify the effectiveness of the proposed method, comparisons were conducted on the KITTI dataset with Faster R-CNN [33], YOLOv3-tiny [34], YOLOv5n [35], YOLOv7-tiny [36], YOLOv8n [37], GOLD-YOLO [38], DINO [16], YOLOv10n [39], YOLOv11n [37], and RT-DETR. All models were evaluated using the same data split, input resolution, and evaluation metrics to ensure the fairness of the experimental comparison. The quantitative comparison results are presented in Table 1.

Table 1 presents the quantitative comparison results of different detection methods on the KITTI dataset. The proposed method achieves a Precision of 93.5%, mAP@0.5 of 95.5%, and mAP@0.5:0.95 of 69.7%, which improve by 3.7%, 1.8%, and 1.1%, respectively, compared with the RT-DETR baseline. The improvement in mAP@0.5 indicates that the proposed method enhances the accuracy of object detection and category recognition. The increase in mAP@0.5:0.95 demonstrates that the proposed method achieves higher overall localization accuracy across multiple IoU thresholds, especially under stricter IoU requirements.

The Recall of the proposed method is 91.1%, which is 1.1% lower than that of RT-DETR, indicating that some challenging objects are still missed during detection. However, considering the overall improvement in Precision and mAP, the proposed model mainly reduces false positives and improves the comprehensive detection performance under different confidence thresholds. Further optimization will be conducted to achieve a better balance between detection accuracy and recall capability.

Regarding model complexity, the number of parameters of the proposed method increases from 30.9 M to 31.8 M, with an increment of only 0.9 M. Meanwhile, the FPS decreases from 95.6 to 92.8, corresponding to a reduction of 2.9%, while still satisfying the requirements of real-time detection. By introducing a small number of additional parameters and incurring a minor speed loss, the proposed method achieves significant improvements in Precision and mAP@0.5, demonstrating a favorable balance between accuracy and efficiency.

To further analyze the detection performance of the proposed method for different categories of traffic objects, the AP values of each category were calculated, and the results are presented in Table 2. Overall, the proposed method achieves performance improvements in five out of the six detection categories, with only a slight decrease in the Van category. Specifically, the AP values of the Car, Truck, Pedestrian, Cyclist, and Tram categories reach 98.1%, 95.4%, 94.2%, 92.0%, and 98.5%, respectively, representing improvements of 1.1, 1.1, 2.1, 1.9, and 5.0 percentage points compared with RT-DETR.

Table 2 summarizes the mAP@0.5 results for each category. Compared with RT-DETR, the proposed method achieves better detection performance in five categories. Among them, the Tram category obtains the most significant improvement, with mAP@0.5 increasing from 93.5% to 98.5%, representing a gain of 5.0 percentage points. The Pedestrian and Cyclist categories improve by 2.1% and 1.9%, respectively, while the Car and Truck categories both achieve an improvement of 1.1%. These results demonstrate that the proposed method improves the detection performance of various traffic objects, particularly for categories that rely heavily on edge, contour, and local feature information.

To reduce the impact of random initialization and training fluctuations on the experimental results, three different random seeds were adopted to independently train RT-DETR and the proposed model. The mean, standard deviation, and 95% confidence interval of each evaluation metric were calculated. As shown in Table 3, the proposed method achieves higher average values than RT-DETR in terms of Precision, mAP@0.5, mAP@0.5:0.95, ${\mathrm{AP}}_{s}$, and ${\mathrm{AR}}_{s}$. Moreover, the standard deviations of all metrics are below 0.4 percentage points, indicating that the model maintains relatively consistent performance across the three independent experiments.

For small-object detection, the ${\mathrm{AP}}_{s}$ of the proposed method increases from 46.8% to 49.5%, representing an improvement of 2.7 percentage points, while ${\mathrm{AR}}_{s}$ increases from 58.1% to 60.4%, with a gain of 2.3 percentage points. These results demonstrate that the proposed method can simultaneously improve the detection accuracy and average recall capability of small objects. Further comparison of the 95% confidence intervals shows that the proposed method achieves overall higher confidence intervals than RT-DETR in terms of Precision, mAP@0.5, mAP@0.5:0.95, and ${\mathrm{AP}}_{s}$, indicating that the performance improvements remain consistent under different random seeds. For ${\mathrm{AR}}_{s}$, the proposed method also obtains a higher mean value and smaller performance fluctuation, further demonstrating the stability of its improvement in small-object recall capability.

To evaluate the adaptability of the proposed method under different data distributions and complex road scenarios, training and testing experiments were conducted on the BDD100K dataset. As shown in Table 4, the proposed method achieves mAP@0.5 and mAP@0.5:0.95 values of 51.7% and 29.7%, respectively, improving by 1.7 and 1.1 percentage points compared with the RT-DETR baseline. These results demonstrate that the proposed frequency-band enhancement and multi-scale fusion strategies remain effective on the BDD100K dataset.

Compared with RT-DETR, as shown in Table 5, the proposed method improves ${\mathrm{AP}}_{s}$ from 14.8% to 16.4%, with an increase of 1.6 percentage points, and improves ${\mathrm{AR}}_{s}$ from 27.9% to 29.6%, with a gain of 1.7 percentage points. This result is consistent with the experimental trend observed on the KITTI dataset, indicating that the proposed frequency-band enhancement strategy can improve small-object detection accuracy and average recall capability to some extent under different data distributions.

Across the three repeated experiments, the proposed method achieves ${\mathrm{mAP}}_{50}$ and ${\mathrm{mAP}}_{50:95}$ values of 51.7±0.2% and 29.7±0.2%, respectively, improving by 1.7 and 1.1 percentage points compared with RT-DETR. The corresponding 95% confidence intervals are overall higher than those of the baseline, indicating that the improvement in overall detection accuracy remains relatively consistent under different random seeds.

### 4.5. Ablation Experiments

To further validate the effectiveness of each proposed improvement module, ablation experiments were conducted based on the RT-DETR baseline model. In these experiments, A denotes the residual enhancement module (RB), which is designed to strengthen the representation of local features in high-frequency subbands. B denotes the high-frequency subband spatial attention module (FA), which adaptively adjusts the responses of different high-frequency information. C denotes the low-frequency cross-scale fusion module, which integrates low-frequency structural information with deep semantic features. Ours represents the complete model incorporating all three components (A + B + C).

As shown in Table 6, after introducing the residual enhancement module A individually, the Precision and ${\mathrm{AP}}_{50}$ of the model are improved by 1.5 and 0.8 percentage points, respectively, indicating that this module enhances the representation of high-frequency detail features and improves object recognition accuracy. The high-frequency subband spatial attention module B and the low-frequency cross-scale fusion module C also bring performance improvements. Among them, module C achieves more significant gains in Precision and ${\mathrm{mAP}}_{50}$ when used individually, demonstrating that the fusion of low-frequency structural information and deep semantic features contributes to improved object representation.

In the module combination experiments, the proposed components exhibit certain synergistic effects. The A+C combination improves ${\mathrm{mAP}}_{50:95}$ by 0.7 percentage points while maintaining a similar Recall. The B+C combination increases ${\mathrm{mAP}}_{50}$ and ${\mathrm{mAP}}_{50:95}$ by 1.6 and 0.9 percentage points, respectively, while preserving the baseline Recall, demonstrating better overall performance. The A+B combination also further improves Precision and ${\mathrm{mAP}}_{50}$, indicating that residual enhancement and frequency attention provide complementary advantages in high-frequency feature modeling.

When all three modules are introduced simultaneously, the complete model achieves the highest performance among all ablation configurations, with Precision, ${\mathrm{mAP}}_{50}$, and ${\mathrm{mAP}}_{50:95}$ reaching 93.5%, 95.5%, and 69.7%, respectively. Compared with the baseline model, these three metrics are improved by 3.7, 1.8, and 1.1 percentage points, respectively. These results demonstrate that high-frequency detail enhancement, frequency attention, and low-frequency cross-scale fusion improve feature representation from different perspectives and further enhance detection accuracy and localization performance when jointly applied.

### 4.6. Visualization Analysis

**Figure 3:**
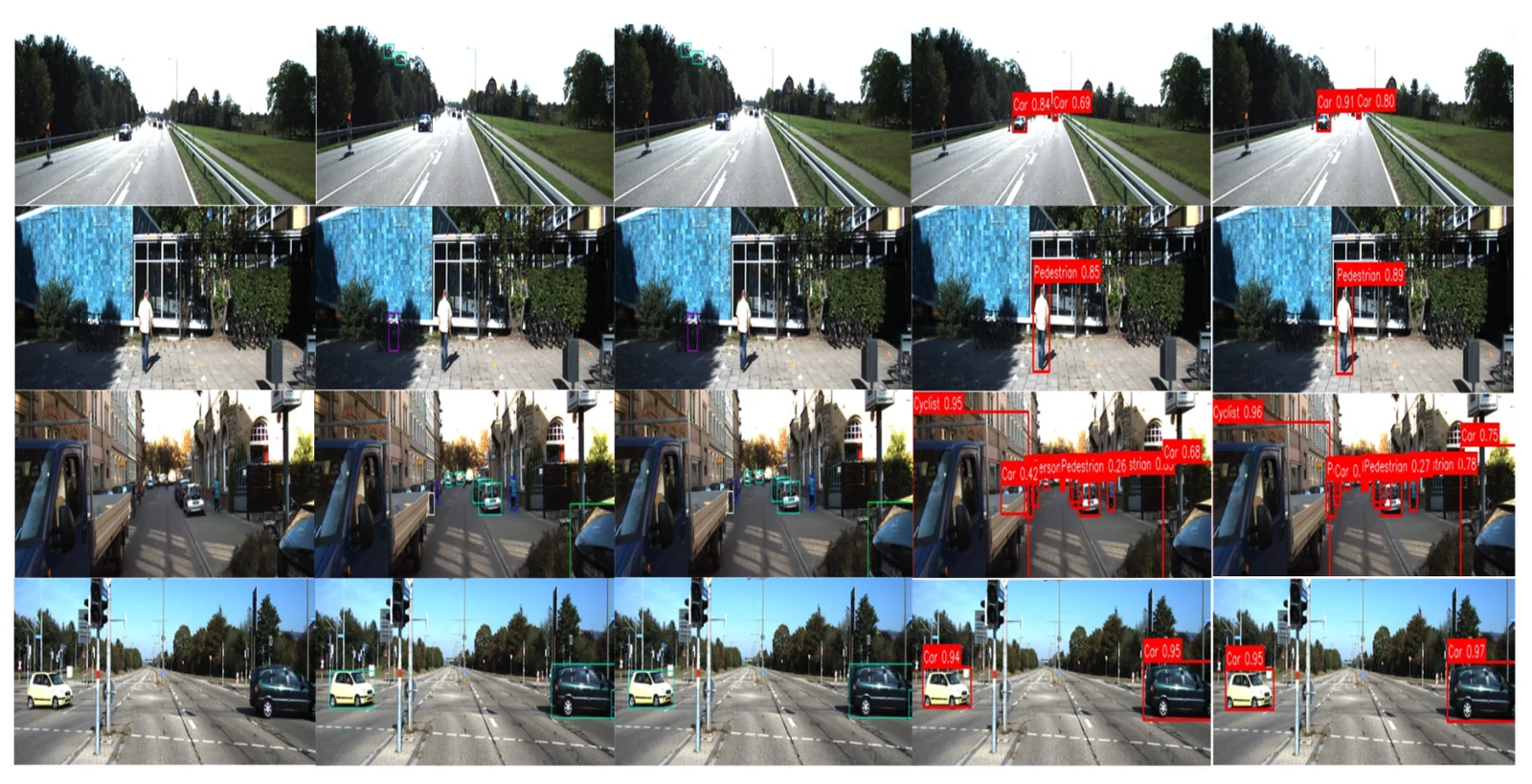
Detection results of different algorithms on the KITTI dataset (from left to right: YOLOv5, YOLOv8, YOLOv10, RT-DETR, and Ours).

To provide a more intuitive comparison of different detection methods, several representative images from the KITTI dataset were selected for visualization. The selected samples contain various road objects, including vehicles, pedestrians, cyclists, and complex background conditions, with the detection results presented in Figure 3. As illustrated in the figure, YOLOv5 and YOLOv8 achieve satisfactory detection performance for nearby and large-scale objects. However, these methods remain prone to missing small targets and heavily occluded objects, particularly in scenes containing dense small-scale objects or complex visual interference.

Benefiting from global feature representation, RT-DETR achieves more stable detection results in complicated road environments and shows stronger capability in identifying distant objects. The visualization results indicate that the proposed method further improves the detection capability for small-scale targets. Nevertheless, a limited number of missed detections still occur in challenging cases involving extremely small objects, severe occlusion, or crowded object distributions. This observation is consistent with the slight reduction in Recall reported in Table 2.

**Figure 4:**
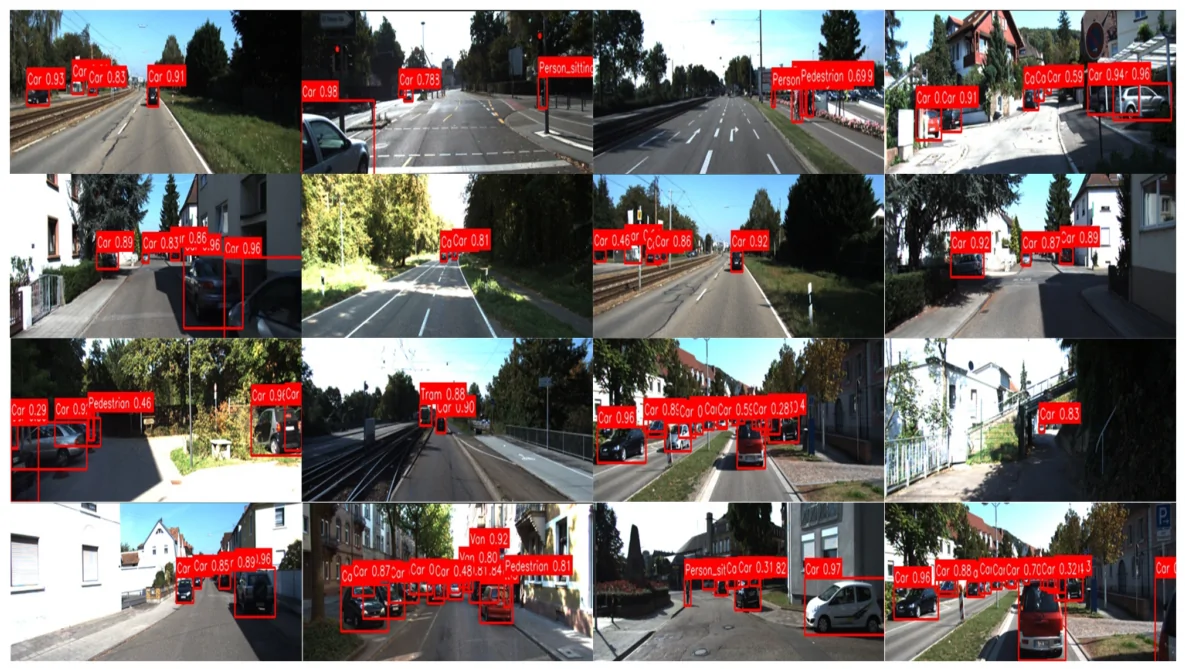
Detection results in different scenarios on the KITTI dataset.

To further evaluate the effectiveness of the proposed detector under different road conditions, Figure 4 presents visualization results from the KITTI dataset. The test samples include diverse driving scenarios, such as urban roads and suburban environments. It can be observed that the proposed method consistently detects major traffic participants, including vehicles, pedestrians, and cyclists, under various conditions. For small-scale objects, the detector is still capable of producing relatively complete detection results, with most objects being correctly classified and the predicted bounding boxes closely matching the actual object locations.

**Figure 5:**
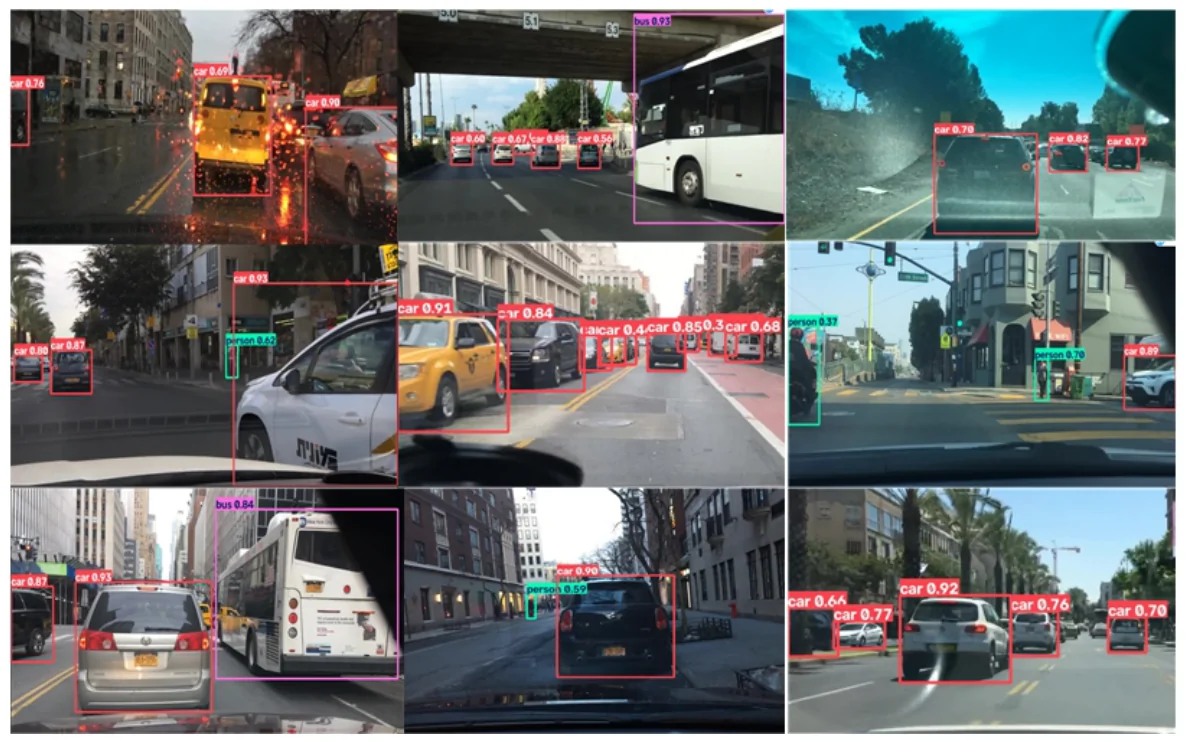
Detection results of different scenarios on the BDD100K dataset.

In addition, Figure 5 provides visualization results on the BDD100K dataset to assess the adaptability of the proposed method in more complex driving scenarios. As shown in the figure, the model can accurately detect vehicles with different scales and maintain reliable performance under dense traffic conditions. Multiple adjacent vehicles can still be distinguished effectively, indicating that the proposed method has a certain degree of robustness against object occlusion and complicated backgrounds.

For distant vehicles and pedestrians with limited pixel resolution, the model is also able to generate appropriate bounding boxes. This suggests that the proposed frequency enhancement and multi-scale fusion strategies effectively preserve edge information and local structural details of small targets. Furthermore, under challenging conditions such as rainy weather, illumination changes, and windshield reflections, the model maintains stable detection performance, demonstrating its potential for practical autonomous driving applications.

## 5. Conclusions

To overcome the limited detection capability of small-scale objects in autonomous driving scenarios, this study presents a frequency-enhanced and multi-scale fusion object detector based on RT-DETR. The proposed approach incorporates a Wavelet Frequency Unit into the cross-scale feature fusion stage of the Neck. Specifically, the input feature maps are decomposed into low-frequency structural information and high-frequency detail components using Haar wavelet transformation. In the high-frequency pathway, a residual enhancement block combined with a spatial attention mechanism is introduced to strengthen object boundaries, texture patterns, and local contour features. Meanwhile, the low-frequency pathway integrates structural information with deeper semantic features through cross-scale fusion, providing additional contextual representations. Finally, an inverse wavelet transformation is applied to reconstruct the enhanced feature maps, thereby improving the feature representation ability of small-scale targets.

Comprehensive experiments on the KITTI dataset demonstrate that the proposed method achieves Precision, AP_50_, and AP_50:95_ values of 93.5%, 95.5%, and 69.7%, respectively, outperforming RT-DETR by 3.7, 1.8, and 1.1 percentage points. Furthermore, AP_s_ and AR_s_ are increased from 46.8% and 58.1% to 49.5% and 60.4%, respectively, indicating that the proposed method can effectively enhance both small-object detection accuracy and recall performance. On the BDD100K dataset, the proposed detector achieves an AP_50_ of 51.7%, while AP_s_ and AR_s_ improve by 1.6 and 1.7 percentage points, respectively. These results demonstrate the effectiveness and adaptability of the proposed method across diverse road environments and data distributions.

Despite the improvements in Precision and average precision, the proposed method still exhibits certain limitations. The overall Recall decreases from 92.2% to 91.1%, suggesting a remaining balance between precision and recall under the current confidence threshold. Additionally, the introduction of the wavelet frequency unit increases the model parameters from 30.9 M to 31.8 M and reduces the inference speed from 95.6 FPS to 92.8 FPS, resulting in additional computational costs. Future research will focus on improving the efficiency of the frequency enhancement module and enhancing the detection capability of challenging targets through multi-scale recall analysis, confidence calibration strategies, and lightweight network optimization. Moreover, further studies will investigate complex scenarios, including adverse weather conditions, severe occlusion, and multimodal information fusion, to improve the robustness and practical deployment capability of autonomous driving systems.

## Figures and Tables

**Table 1:** Performance comparison of different object detection methods on the KITTI test set.

Model	P (%)	R (%)	mAP@0.5 (%)	mAP@0.5:0.95 (%)	Parameters (M)	FPS
Faster-RCNN	82.6	73.4	72.8	43.0	41.3	18.7
YOLOv3-tiny	85.1	72.2	75.7	53.0	8.5	154.7
YOLOv5n	87.0	77.5	84.0	53.2	1.9	125.4
YOLOv7-tiny	88.9	79.0	86.8	45.1	6.2	104.7
YOLOv8n	89.2	80.7	87.5	57.3	3.1	135.0
GOLD-YOLO	90.8	81.6	88.6	59.8	35.6	118.4
DINO	89.5	81.2	88.7	59.4	23.0	87.2
YOLOv10n	90.4	81.6	88.5	60.8	2.3	126.3
YOLOv11n	91.8	82.0	89.5	61.5	2.6	134.5
RT-DETR	89.8 ± 0.3	92.2 ± 0.2	93.7 ± 0.2	68.6 ± 0.2	30.9	95.6
Ours	93.5 ± 0.2	91.1 ± 0.3	95.5 ± 0.2	69.7 ± 0.2	31.8	92.8

**Table 2:** Detection performance comparison of different methods on different categories.

Model	Car	Van	Truck	Pedestrian	Cyclist	Tram
Faster-RCNN	81.3	72.0	68.5	70.5	73.8	70.7
YOLOv3-tiny	82.8	76.0	72.5	72.5	75.4	75.0
YOLOv5n	91.5	81.8	79.2	85.3	86.6	79.6
YOLOv7-tiny	94.2	84.7	81.3	88.8	88.1	83.7
YOLOv8n	95.1	85.3	82.4	90.2	89.4	82.6
GOLD-YOLO	96.2	86.8	83.5	91.2	90.4	83.5
DINO	96.8	86.1	82.9	91.8	90.7	83.9
YOLOv10n	97.1	85.6	82.1	91.6	90.8	83.3
YOLOv11n	97.4	87.0	83.8	92.2	91.1	85.5
RT-DETR	97.0	95.2	94.3	92.1	90.1	93.5
Ours	98.1	94.8	95.4	94.2	92.0	98.5

**Table 3:** Statistical results of repeated experiments between RT-DETR and the proposed method on the KITTI dataset.

Metric	RT-DETR (Mean ± SD)	Ours (Mean ± SD)	95% CI of RT-DETR	95% CI of Ours
P (%)	89.8 ± 0.3	93.5 ± 0.2	[89.05, 90.55]	[93.00, 94.00]
R (%)	92.2 ± 0.2	91.1 ± 0.3	[91.70, 92.70]	[90.35, 91.85]
mAP@0.5 (%)	93.7 ± 0.2	95.5 ± 0.2	[93.20, 94.20]	[95.00, 96.00]
mAP@0.5:0.95 (%)	68.6 ± 0.2	69.7 ± 0.2	[68.10, 69.10]	[69.20, 70.20]
AP_s_ (%)	46.8 ± 0.4	49.5 ± 0.3	[45.81, 47.79]	[48.76, 50.24]
AR_s_ (%)	58.1 ± 0.3	60.4 ± 0.2	[57.36, 58.84]	[59.90, 60.90]

**Table 4:** Performance comparison of different methods on the BDD100K dataset.

Model	Parameters (M)	FPS	mAP@0.5 (%)	mAP@0.5:0.95 (%)
Faster-RCNN	41.3	18.7	47.9	26.5
YOLOv3-tiny	8.5	154.0	46.7	25.9
YOLOv5n	1.9	125.4	49.5	26.2
YOLOv7-tiny	6.2	104.7	50.7	29.1
YOLOv8n	3.1	135.0	51.2	28.7
RT-DETR	30.9	95.6	50.0	28.6
Ours	31.8	92.8	51.7	29.7

**Table 5:** Statistical results of repeated experiments between RT-DETR and the proposed method on the BDD100K dataset.

Metric	RT-DETR (Mean ± SD)	Ours (Mean ± SD)	95% CI of RT-DETR	95% CI of Ours
${\mathrm{mAP}}_{50}\left(\%\right)$	50.0 ± 0.3	51.7 ± 0.2	[49.25, 50.75]	[51.20, 52.20]
${\mathrm{mAP}}_{50:95}\left(\%\right)$	28.6 ± 0.2	29.7 ± 0.2	[28.10, 29.10]	[29.20, 30.20]
${\mathrm{AP}}_{s}\left(\%\right)$	14.8 ± 0.3	16.4 ± 0.3	[14.05, 15.55]	[15.65, 17.15]
${\mathrm{AR}}_{s}\left(\%\right)$	27.9 ± 0.4	29.6 ± 0.3	[26.91, 28.89]	[28.85, 30.35]

**Table 6:** Ablation study results of the proposed method on the KITTI dataset.

Model	P (%)	R (%)	mAP@0.5 (%)	mAP@0.5:0.95 (%)
RT-DETR	89.8	92.2	93.7	68.6
RT-DETR+A	91.3	91.8	94.5	68.4
RT-DETR+B	92.1	91.6	94.8	69.2
RT-DETR+C	92.8	91.4	95.1	69.0
RT-DETR+A+B	92.5	91.6	95.0	68.8
RT-DETR+A+C	93.1	92.1	95.1	69.3
RT-DETR+B+C	92.9	92.2	95.3	69.5
Ours	93.5	91.1	95.5	69.7

## Data Availability

The original contributions presented in this study are included in the article. Further inquiries can be directed to the corresponding author.
